# Clinical impact of potential drug-drug interactions between midostaurin and posaconazole in FLT3-mutated AML

**DOI:** 10.1128/aac.01951-25

**Published:** 2026-05-12

**Authors:** Carolin S. Joisten, Sibylle C. Mellinghoff, Danila Seidel, Carsten Müller, Charlotte Müller-Ohrem, Karl-Anton Kreuzer, Lukas P. Frenzel, Florian Simon, Michael Hallek, Philipp Koehler, Oliver A. Cornely, Jannik Stemler

**Affiliations:** 1 Department I of Internal Medicine, Center for Integrated Oncology (CIO), Medical Faculty and University Hospital of Cologne, University of Cologne 14309, Cologne, Germany; 2 Faculty of Medicine and University Hospital Cologne, Institute of Translational Research, University of Cologne, Cologne Excellence Cluster on Cellular Stress Responses in Aging-Associated Diseases (CECAD) 14309, Cologne, Germany; 3 German Center for Infection Research (DZIF), Partner Site Bonn-Cologne, Cologne, Germany; 4 TDM Laboratory, Center for Laboratory Diagnostics, University Hospital of Cologne, Cologne, Germany; 5 Department I of Internal Medicine, Division of Clinical Immunology, Medical Faculty and University Hospital of Cologne, University of Cologne 14309, Cologne, Germany; 6 Clinical Trials Center Cologne (ZKS Köln), University of Cologne, Faculty of Medicine and University Hospital Cologne 14309, Cologne, Germany; University Children's Hospital Münster, Münster, Germany

**Keywords:** adverse effects, targeted therapy, cytochrome p450, therapeutic drug monitoring, antifungal prophylaxis, aspergillosis, mucormycosis, leukemia

## Abstract

To determine midostaurin and posaconazole plasma concentrations and investigate adverse events (AEs) resembling drug-drug interactions (DDI) when both drugs were administered concomitantly during induction chemotherapy for acute myeloid leukemia (AML). Patients with FLT3-mutated AML who received midostaurin and posaconazole concomitantly between May 2019 and December 2022 were included and followed up to March 2023. Twice-weekly trough levels for midostaurin and posaconazole were measured with validated liquid chromatography–tandem mass spectrometry methods. Potential DDIs were independently reviewed by two physicians and attributed using the Drug Interaction Probability Scale (DIPS). Population pharmacokinetics analysis was done via nonlinear mixed-effect modeling. In 29 patients, concentrations ranged from 0.6 to 24.5 mg/L for midostaurin and from <30 to 2,572 µg/L for posaconazole. A total of 375 AEs in 66 midostaurin cycles, with 280 AEs classified as grade ≥3, were recorded. Probable DDI with a DIPS score of ≥5 was attributed in 14/375 AEs; no highly probable AEs were registered. Eight AEs led to dose modification or discontinuation of midostaurin in seven patients. Clearance for midostaurin during co-administration with posaconazole was 0.52 L/h (95% CI, 0.42–0.62 L/h). A breakthrough fungal infection was recorded in eight patients (27.5%). DDI of midostaurin and posaconazole is clinically meaningful but infrequent. High inter- and intra-individual variabilities of midostaurin and posaconazole plasma exposure were observed. Midostaurin clearance was delayed during co-administration. Midostaurin therapeutic drug monitoring may serve for decision-making when DDI with CYP3A4 inhibitors is suspected.

## INTRODUCTION

Midostaurin is a multikinase inhibitor to treat *FLT3*-mutated acute myeloid leukemia (AML). It was approved in the European Union in 2019 following the results of the randomized controlled RATIFY trial, which demonstrated improved overall survival in patients treated with a combination of midostaurin and intensive remission-induction chemotherapy (RIC) compared to RIC alone (1).

Antifungal prophylaxis with mold-active triazoles, preferably posaconazole, to prevent invasive fungal diseases (IFDs) is strongly recommended in patients with AML during RIC as standard of care due to its benefit in improving survival (2–4).

However, since midostaurin is metabolized by the cytochrome P450 system, particularly via CYP3A4, and posaconazole inhibits this enzyme, coadministration increases the risk of drug-drug interactions (DDIs). Due to these concerns, it has become common practice to withhold triazoles, despite other antifungal strategies being inferior in preventing IFD and death (5, 6).

Therapeutic drug monitoring (TDM) may be a promising tool to monitor drug levels and correlate potential DDI within the clinical context, as well as to guide dosing and optimize treatment.

Here, we report a cohort of patients with AML from a tertiary care hematology center who received concomitant posaconazole and midostaurin, with a focus on clinically relevant adverse events (AEs) resembling potential DDI, including TDM of plasma trough levels for both drugs.

## MATERIALS AND METHODS

### Study design and population

Patients with *FLT3*-mutated AML treated at the University Hospital Cologne in Germany who received concomitant midostaurin and posaconazole were included in a prospective, observational, explorative study between May 2019 and December 2022 and followed up to March 2023. Written informed consent was obtained from each patient (Study ID: 08-160 and 13-091). Standard RIC in patients with AML fit for intensive treatment consisted of at least one cycle of “3 + 7” induction chemotherapy, while until January 2021, two cycles of “3 + 7” were a standard. In patients with CD33+ AML, gemtuzumab-ozogamicin was added. Consolidation included high-dose or intermediate-dose cytarabine. As a standard of care, all patients with AML received posaconazole prophylaxis, with 300 mg oral delayed-release tablets, during chemotherapy and episodes of neutropenia, making a control group not feasible (4). Trough concentrations of both drugs were measured twice weekly with a validated heated electrospray ionization liquid chromatography–tandem mass spectrometry (HESI-LC-MS/MS) method as described previously (7, 8) (Fig. S1).

### Definitions

Severe neutropenia was defined as an absolute neutrophil count of <0.5 × 10e9/L. Posaconazole plasma level >700 µg/L was considered effective for prophylaxis (9). For midostaurin, reference ranges are still pending to be determined. IFD was defined according to the EORTC/MSG 2020 criteria (10).

### Data documentation

Clinical data of all patients were collected in an online electronic case report form on https://www.clinicalsurveys.net/ (EFS Summer 2021, TIVIAN GmbH, Cologne, Germany) and collected basic demographic data, hematological diagnosis, comorbidities, treatment regimen of the underlying disease, diagnostics for *FLT3*-mutation, serum hematological and chemical parameters of electrolytes and kidney and liver function, antifungal drugs for prophylaxis, other concomitant medication and blood levels via TDM, if available, adverse events, and, as events of special interest, the incidence of IFD; and outcome after 30 and 90 days.

### Adverse events

AEs were defined and documented according to the CTCAE v5.0 (Common Terminology Criteria for Adverse Events) into mild, moderate, severe, life-threatening, and death related to AE from the first day of midostaurin treatment, i.e., when posaconazole was already dosed (11). All AEs occurring during and up to 7 days after stopping concomitant medication of midostaurin and posaconazole were recorded and attributed or not attributed to either one of the drugs and/or a potential DDI, also considering their respective Summary of Product Characteristics by two physicians (JS and SCM) (12, 13). Time to onset of adverse events was defined as the time between the first dose of simultaneous administration of midostaurin and posaconazole to the first occurrence of AE of interest.

### Clinical drug-drug interactions

Potential DDIs between midostaurin and posaconazole were defined as the primary outcome. The Drug Interaction Probability Scale (DIPS) was calculated for all documented AEs suspected to be due to DDI and reviewed by two physicians (JS and SCM) (14). The DIPS scores potential DDI according to a 10-item questionnaire including consistencies with known interactive properties, time courses, and evaluation of alternative causes, and classifies a DDI as “doubtful” (score <2), “possible” (score 2–4), “probable” (5–8), and “highly probable” (score >8). Clinically notable AEs (CNAEs) were defined as cardiac AEs, any infections, and all AEs that led to treatment interruption, dose modification, or discontinuation, similar to the analyses of the manufacturer (15). Incidence and characteristics of AEs were analyzed in the study population and correlated with drug plasma levels of both midostaurin and posaconazole.

### Analysis of midostaurin and posaconazole plasma levels

Midostaurin and posaconazole concentrations were measured in blood plasma based on validated HESI-LC-MS-MS assays, in accordance with international validation guidelines (7, 8). Plasma levels of both midostaurin and posaconazole were analyzed descriptively. Other concomitant medications influencing the metabolization of midostaurin, i.e., other CYP3A4 inhibitors, were recorded as well.

### Population pharmacokinetics analysis

The time-concentration profile of this real-life population was best described by a two-compartment model, and the following pharmacokinetic parameters were calculated via nonlinear mixed-effect modeling (NONMEM VII): mean apparent serum clearance: CL/*F* (L/h), volume of distribution in the central compartment: *V*_*c*_/*F* (L), volume of distribution in the peripheral compartment: *V*_*p*_/*F* (L), absorption constant: *K*_*a*_ (1/h), and intercompartmental clearance: *Q* (L/h).

### Statistical analysis

Statistical analysis was performed with Microsoft Excel 2019 MSO (version 2311 Build 16.0.17029.20028) and with IBM SPSS v.28.0 (IBM, Chicago, USA; RRID:SCR_016479). The figures were created using GraphPad Prism (version 10.4.1; RRID:SCR_002798) and RStudio (version 2022.07.1+554; RRID:SCR_000432).

## RESULTS

In total, 29 patients were included in the final analysis after exclusion of patients who had not received midostaurin and posaconazole concomitantly, patients with missing TDM, and patients who were lost to follow-up (Fig. S2).

Demographic, clinical characteristics, and outcome are presented in Table 1. The median age of the patients at the time of diagnosis of AML in our study cohort was 47 years (range 18–74, interquartile range [IQR] 34–63). The median duration of in-hospital treatment during induction chemotherapy was 44 days (range 25–140, IQR 35–65), during which 18 patients were treated on the intensive care unit or intermediate care for a median duration of 4 days (range 0–54, IQR 0–9). During the inpatient stay for RIC, the median length of severe neutropenia was 19 days (range 5–61, IQR 16–25).

**TABLE 1:** Patient characteristics^*a*^

*n* = 29	*n* (%)
Sex	
Female	13 (44.8)
Male	16 (55.2)
Median age, years (IQR)	47 (34-63)
ELN classification^*b*^ (16)	
Favorable	12 (41.4)
Intermediate	10 (34.5)
Adverse	7 (24.1)
Chemotherapy regimen^*c*^	
Induction 7 + 3 (cytarabine/anthracycline) with midostaurin	41 (60.3)
Consolidation of high-dose cytarabine and midostaurin	25 (36. 8)
Maintenance treatment with midostaurin^*d*^	2 (2.9)
Incidence of bIFD based on 2020 EORTC/MSG criteria	8 (100.0)
Possible	3 (37.5)
Probable	4 (50.0)
Proven	1 (12.5)
Complete remission of AML	
Yes	21 (72.4)
No	5 (17.2)
Unknown	3 (10.3)
Survival	
Overall^*e*^	17 (58.6)
30 days	28 (96.6)
90 days	27 (93.1)

^*a*^ AML, acute myeloid leukemia; bIFD, breakthrough invasive fungal disease; ELN, European Leukemia Network; IFD, invasive fungal disease; IQR, interquartile range.

^*b*^ All patients had a diagnosis of *FLT3*mut AML.

^*c*^ Only chemotherapy cycles in which midostaurin and posaconazole were given concomitantly.

^*d*^ Maintenance treatment from days 8 to 365, in two patients with secondary antifungal prophylaxis with posaconazole; these patients were excluded from adverse events analysis.

^*e*^ Median observation period of 270 days (range 19–1,169, IQR 152–624).

**Fig 1:**
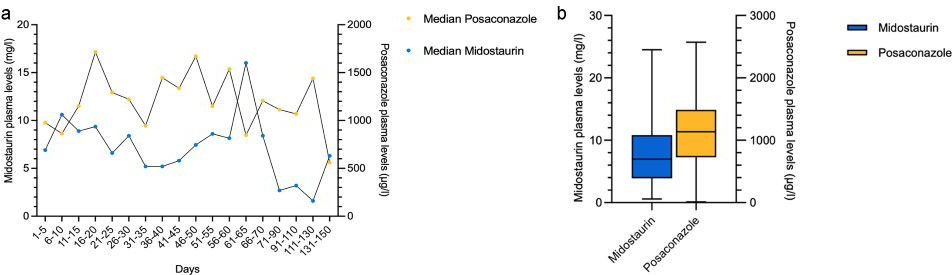
(**a**) Midostaurin and posaconazole plasma levels of all patients—median over the time. (**b**) Midostaurin and posaconazole plasma levels in 29 patients.

In patients who received midostaurin and posaconazole concomitantly at any time, the median total duration of treatment was 14 days (range 1–341, IQR 12–14) and 77 days (range 6–379, IQR 49–149), respectively (Table 2). Midostaurin and posaconazole were prescribed concomitantly for a median of 14 days (range 1–52, IQR 9–14), including midostaurin maintenance therapy. Inter- and intra-individual variability of midostaurin and posaconazole plasma exposure is depicted in Fig. S3. The median plasma concentration of midostaurin was 7.0 mg/L (range 0.6–24.5 mg/L, IQR 3.9–10.8 mg/L), the mean 7.86 mg/L (standard deviation [SD], ±5.28 mg/L), and the geometric mean 6.0 mg/L (SD, ±2.22 mg/L). For posaconazole, the median of plasma concentration measured 1,135 µg/L (range <30–2,572 µg/L, IQR 738–1,485 µg/L), the mean 1,163 µg/L (SD, ±559 µg/L), and the geometric mean 978 µg/L (SD, ±2 µg/L; Fig. 1b). Median midostaurin and posaconazole plasma levels over time are depicted in Fig. 1a.

**TABLE 2:** Administration of midostaurin and posaconazole^*a*^

	*n* (%)
Midostaurin	
Median duration of administration, days (IQR)	14 (12–14)
Dose, *n* (%)	
25 mg b.i.d.	1 (1.2)
50 mg b.i.d.	13 (16.0)
100 mg b.i.d.	67 (82.7)
Posaconazole	
Median duration of administration, days (IQR)	76.5 (49.0–148.8)
Dose, *n* (%)	
200 mg q.d.	6 (10.7)
300 mg q.d.^*b*^	45 (80.3)
400 mg q.d.	3 (5.4)
500 mg q.d.	1 (1.8)
600 mg q.d.	1 (1.8)
Median of simultaneous intake of midostaurin and posaconazole, days (IQR)	14 (9–14)
Other concomitant CYP3A4 inhibitors during the administration of midostaurin, *n* (%)	
Voriconazole	2 (6.9)
Ciprofloxacin	4 (13.8)
Clarithromycin	1 (3.4)
None	22 (79.3)

^*a*^ b.i.d., two times a day; IQR, interquartile range; q.d., once a day.

^*b*^ All patients were started on 300 mg of posaconazole on day 1 or earlier of RIC and on midostaurin on day 8 of RIC.

Several patients underwent midostaurin dose adaptations. Within one midostaurin administration cycle, the standard dose of 100 mg per day was decreased to 50 mg per day in six patients on days 13, 21, 29, 45, 57, and 63, respectively, and reduced from 50 mg to 25 mg in one patient on day 57. The clinical rationale for dose changes was dose adaptations according to suspected toxicities based on TDM results and clinical presentations. The median plasma concentration of midostaurin in three patients with 13 measured plasma concentrations before dose adaptations was 10 mg/L (range 5.2–15.7 mg/L, IQR 7.4–12 mg/L), which was higher than the median plasma concentration of midostaurin of 7 mg/L in the full cohort.

In patients receiving posaconazole, dose adaptations were performed as follows: dose reductions included from 300 mg tablet to 200 mg tablet (*n* = 4) and from 400 mg tablet to 300 mg tablet (*n* = 1). Due to insufficient target levels for prophylactic efficacy, the dose for posaconazole was increased from 300 mg tablet to 400 mg tablet (*n* = 3) and from 400 mg tablet to 500 mg tablet (*n* = 1). In three patients, the administration was changed from tablet to oral suspension during prophylaxis due to gastrointestinal alterations (mucositis, proton pump inhibitor administration); the dosage of posaconazole was changed from 300 mg tablet to 200 mg t.i.d. oral suspension (*n* = 2) and from 300 mg tablet to 600 mg b.i.d. oral suspension (*n* = 1).

Seven patients (20.7%) received other CYP3A4 inhibitors bearing the risk for DDI in addition to midostaurin: Ciprofloxacin was prescribed at the same time as midostaurin in four (13.7%) patients, in three cases as prophylaxis and in one case as treatment for *Klebsiella pneumoniae* infection. The strong CYP3A4 inhibitors voriconazole for fungal pneumonia (2/29 [6.9%]), when posaconazole was paused, and clarithromycin for community-acquired pneumonia (1/29 [3.5%]) were simultaneously administered to midostaurin in three patients (Table S1).

### Analysis of adverse events and potential DDI

DDI analyses were performed in all subjects who received concomitant midostaurin and posaconazole during RIC or consolidation for at least 1 day. The maintenance cycles were excluded from this analysis as concomitant administration of midostaurin and posaconazole was not done in this setting.

Overall, 375 AEs in 66 cycles of midostaurin with a mean time to onset of 4.4 days (SD ±6.5 days) were recorded. Excluding expected hematological toxicity-associated AEs (*n* = 174), in particular anemia (*n* = 60), thrombocytopenia (*n* = 58), and leukocytopenia (*n* = 56), a total of 201 AEs were recorded (Table 3). At the initiation of midostaurin treatment, 133/375 (35.5%) AEs were already present. All other AEs (242/375, 64.5%) had a mean time of onset of 6.8 days (SD, ±7.0 days; Table 3).

**TABLE 3:** Adverse events during concomitant treatment with midostaurin and posaconazole^*a*^^,^^*b*^

	All grades *n* (%)	Grade 3/4/5 *n* (%)
Any	375 (100.0)	280 (100.0)
Hematological AEs	174 (46.4)	160 (57.1)
Leukocytopenia	56 (14.9)	54 (19.3)
Thrombocytopenia	58 (15.5)	58 (20.7)
Anemia	60 (16.0)	48 (17.1)
Febrile neutropenia	51 (13.6)	50 (17.9)
Gastrointestinal AEs	34 (9.0)	5 (0.8)
Diarrhea	14 (3.7)	0 (0.0)
Constipation	1 (0.3)	0 (0.0)
Nausea	12 (3.2)	0 (0.0)
Mucositis/neutropenic enterocolitis	6 (1.6)	5 (1.8)
Ileitis terminalis	1 (0.3)	0 (0.0)
Infections^*c*^	47 (12.5)	45 (16.1)
Device-related infection	4 (1.1)	4 (1.4)
Upper respiratory tract infection	2 (0.5)	2 (0.7)
Pneumonia	10 (2.7)	10 (3.6)
Fungal infection^*d*^	8 (2.1)	8 (2.9)
Bacteremia	18 (4.8)	17 (6.1)
Viremia	1 (0.3)	1 (0.4)
Herpes simplex infection	4 (1.1)	4 (1.4)
*Clostridioides difficile* infection	1 (0.3)	1 (0.4)
Wound infection	1 (0.3)	1 (0.4)
Neutropenic sepsis	7 (1.9)	6 (2.1)
Septic shock	1 (0.3)	1 (0.4)
Pulmonary AEs	4 (1.1)	2 (0.7)
Pulmonary edema	2 (0.5)	2 (0.7)
Pleural effusion	1 (0.3)	0 (0.0)
Atelectasis	1 (0.3)	0 (0.0)
Cardiac AEs	16 (4.3)	8 (2.9)
QTc prolongation^*e*^	6 (1.6)	2 (0.7)
Bradycardia	1 (0.3)	1 (0.4)
Hypertension	1 (0.3)	1 (0.4)
Troponinemia	2 (0.5)	1 (0.4)
Pericardial effusion	2 (0.5)	0 (0.0)
Hydropic decompensation	1 (0.3)	1 (0.4)
Chest pain	1 (0.3)	0 (0.0)
Perimyocarditis	1 (0.3)	1 (0.4)
Sudden cardiac death	1 (0.3)	1 (0.4)
Laboratory abnormalities^*f*^	26 (6.9)	4 (1.4)
Acute liver failure	1 (0.3)	1 (0.4)
Acute kidney failure	1 (0.3)	0 (0.0)
Urinary tract obstruction	1 (0.3)	0 (0.0)
Neurological AEs	4 (1.1)	0 (0.4)
Vascular AEs	1 (0.3)	1 (0.4)
Bleeding AEs	3 (0.8)	3 (1.1)
Skin and subcutaneous tissue AEs	10 (2.7)	1 (0.4)
Ophthalmological AEs	2 (0.5)	0 (0.0)

^*a*^ 12.9 (375/29) AEs occurred per patient, 5.7 (375/66) AEs occurred per midostaurin cycle 66 cycles of midostaurin concomitant to posaconazole prophylaxis, excluding maintenance treatment: 29 patients received a first cycle (C1), 18 patients a second cycle (C2), 11 patients a third cycle (C3), 7 patients a fourth cycle (C4), and 1 patient a fifth cycle (C5).

^*b*^ AEs, adverse events.

^*c*^ Numbers may overlap.

^*d*^ Proven, probable, or possible IFD according to the 2020 EORTC/MSG criteria.

^*e*^ We performed a baseline electrocardiogram (ECG) before administration of midostaurin, weekly during the administration of midostaurin, and when toxicity was suspected. The median of baseline QTcB when AML was diagnosed was 420 ms. During the administration of midostaurin, the median of QTcB was slightly higher at 435 ms. In five patients, no baseline ECG before administration of midostaurin was performed.

^*f*^ Bilirubin, elevated alanine aminotransferase, hypokalemia, hyponatremia, and hypernatremia.

The number of AEs of grade ≥3 was 280/375 (74.6%). When hematological toxicity-associated AEs were excluded, 120/375 (32.0%) AEs were grade ≥3. Of the AEs grade ≥3, 120/280 (42.9%) were present at the beginning of midostaurin treatment. All other AEs grade ≥3 (160/280, 57.1%), which were not present at the administration start of midostaurin, had a mean time of onset of 6.8 days (SD, ±7.5 days; Table 1).

CNAEs graded ≥3 were 95/375 (25.1%), excluding CNAEs present at the start of midostaurin. The mean time to onset of CNAEs grade ≥3 was 9.1 days (SD, ±8.8 days).

From the total 375 AEs, 301 (80.3%) AEs were DIPS score <2, while 74 (19.7%) AEs were DIPS score ≥2. Only 14/375 (3.7%) AEs had a DIPS score of ≥5, meaning that the DDI was probable (Table 4). No highly probable DIPS score >8 was recorded. In patients with AEs and a DIPS score of ≥5, median trough plasma levels of midostaurin were 7.4 mg/L (range 1.8–19.5 mg/L, IQR 6.1–11.3 mg/L) and of posaconazole 892 µg/L (range 274–1,686 µg/L, IQR 591–1,099 µg/L) before, at, and after the onset of the AE.

**TABLE 4:** Adverse events with probable (DIPS score ≥5) drug-drug interaction attribution (*n* = 29 patients, *n* = 66 midostaurin cycles)^*a*^^,^^*b*^^,^^*c*^

Adverse event (AE)	DIPS Score	Description	Posaconazole trough level (µg/L)	Midostaurin trough level (mg/L)	Comment
QTc prolongation and bradycardia Two AEs in one patient	8	Symptomatic sinus bradycardia (38bpm) and QTcB prolongation of 520ms 6 days after start of concomitant intake of midostaurin and posaconazole. Vasopressors needed, ICU transfer.	627 (1 day before onset of AE)	10.2 (1 day before onset of AE)	No other cause identified. Attributed to midostaurin, due to recovery of bradycardia and QTc prolongation after discontinuation of midostaurin and posaconazole.
Perimyocarditis One AE in one patient	5	Chest pain on day 5 after midostaurin intake. Suspected myocardial infarction or pulmonary embolism ruled out, ICU transfer. Cardiac MRI: hypocinesia of LV, myocardial edema, myocardial viruses negative.	662 (1 day before onset of AE)	5.5 (1 day before onset of AE)	Other causes for perimyocarditis ruled out. Attributed to midostaurin; spontaneous recovery after discontinuation of midostaurin three days after onset of AE.
Pericardial effusion Two AEs in two patients	5	Pericardial effusion of 5mm without haemo-dynamic relevance in TTE	892 (on the day of onset of AE)	7.1 (on the day of onset of AE)	No other cause identified; attributed to midostaurin.
		Pericardial effusion of 5 mm in TTE	1,047 (4 days before onset of AE) 914 (on the day of onset of AE)	7.0 (4 days before onset of AE) 9.3 (on the day of onset of AE)	No other cause identified; attributed to midostaurin.
QTc prolongation Five AEs in three patients	5	Patient 1: First cycle of midostaurin QTcB of 488ms after 6 days of simultaneously intake of midostaurin (100mg) and posaconazole.	921 (on the day of onset of AE)	Not done	No other causes identified, most likely attributed to midostaurin. Dosage of midostaurin was reduced by 50%; two days later, the patient had a normal QTcB of 427ms.
		Patient 1: Second cycle of midostaurin QTcB of 480ms after 44 days of simultaneously intake of midostaurin (25mg) and posaconazole.	431 (on the day of onset of AE)	16.0 (on the day of onset of AE)	No other causes identified, most likely attributed to midostaurin. Midostaurin was discontinued.
		First cycle of midostaurin QTcB of 478ms after 11 days of simultaneously intake of midostaurin and posaconazole.	1,603 (2 days after onset of AE) 1,686 (9 days after onset of AE)	15.0 (2 days after onset of AE) 7.4 (9 days after onset of AE)	No other causes identified, most likely attributed to midostaurin. Dosage of midostaurin was discontinued for nine days and then continued with a dose reduction by 50% due to a still long QTcB of 482ms (one day before second cycle).
		QTcB of 485ms after 16 days of simultaneously intake of midostaurin and posaconazole.	1,151 (1 day before onset of AE) 892 (1 day after onset of AE)	19.5 (1 day before onset of AE) 7.1 (1 day after onset of AE)	No other causes identified, most likely attributed to midostaurin. The patient received midostaurin till two days before onset of AE.
Hypokalemia Five AEs in three patients	5	Decrease for 9 days; started 3 days after simultaneously intake of midostaurin and posaconazole.	361 (3 day after onset of AE) 274 (7 days after onset of AE) 555 (10 days after onset of AE)	12.4 (3 days after onset of AE) 9.4 (7 days after onset of AE) 5.2 (10 days after onset of AE)	No other causes identified, most likely attributed to midostaurin. No discontinuation or interruption of midostaurin during occurrence of AE.
		Decrease for one day; started 5 days after simultaneously intake of midostaurin and posaconazole.	Not done	Not done	No other causes identified, most likely attributed to midostaurin. No discontinuation or interruption of midostaurin during occurrence of AE.
		Decrease for 1 day; started 1 day after simultaneously intake of midostaurin and posaconazole.	1,213 (on the day of onset of AE)	1.8 (on the day of onset of AE)	No other causes identified, most likely attributed to midostaurin. No discontinuation or interruption of midostaurin during occurrence of AE.

^*a*^ Explanation DIPS score: “doubtful” (score <2), “possible” (score 2–4), “probable” (5–8), and “highly probable” (score >8).

^*b*^ DIPS score for 301/375 (80.3%) was <2 (drug-drug interaction doubtful), and no highly probable AEs with a DIPS score >8 were recorded.

^*c*^ AEs, adverse events; DIPS, Drug Interaction Probability Scale; ICU, intensive care unit; LV, left ventricle; MRI, magnetic resonance imaging; Pip/Taz, piperacillin/tazobactam; QTcB, corrected QT interval after Bazett; TTE, transthoracic echocardiography.

Of the 95 CNAEs grade ≥3, 20 (21.1%) had a DIPS score ≥2. The number of infections grade ≥3 was 82/375 (21.9%; Table S2). In seven patients, eight CNAEs (grade ≥3) led to dose modification, treatment interruption, or discontinuation of midostaurin. The responsible AEs were dermatitis, liver failure, lung infection, mucositis, neutropenic enterocolitis, bradycardia, and QTc prolongation (2×). None of these were fatal.

### Population pharmacokinetics analysis

The calculated ﬁnal clearance parameter estimate for midostaurin was as follows: clearance (Cl) = 0.52 L/h (95% CI, 0.42–0.62 L/h) and *K*_*a*_ = 0.0304 (95% CI, 0.027–0.088), with a remaining interindividual variability in Cl characterized by a coefficient of variation of 47.1%. Clearance was influenced by body weight and showed an age-dependent change. A Goodness-of-fit plot for population pharmacokinetic analysis is shown in Fig. S4.

### Incidence of breakthrough IFD

Incidence of breakthrough IFD (bIFD) during posaconazole prophylaxis was 8/29 (27.5%). Three cases were possible (37.5%), four probable (50.0%), and one proven (12.5%) according to the revised 2020 EORTC/MSG criteria (Table 1; Table S3). Concomitant medication with midostaurin and posaconazole was administered in 5/8 (62.5%) patients with bIFD, with a median time of onset from diagnosis of AML to bIFD of 73 days (range 20–100, IQR 50–88; 2/5 diagnosed during induction; 3/5 diagnosed during consolidation). Of these five patients, two patients had low posaconazole plasma levels and 2.57- and 1.21-fold higher midostaurin plasma levels compared to our study population before IFD was diagnosed, respectively. Two patients had therapeutic posaconazole concentrations and compared to our study population 2.04- and 1.96-fold lower midostaurin plasma levels before development of IFD (Fig. S5).

### Outcome

The 30- and 90-day survival rates after diagnosis of AML were 96.6% (28/29) and 93.1% (27/29), respectively. After the median observation period of 270 days (range 19–1,169, IQR 152–624), overall survival was 58.6% (17/29). Mortality attributed to bIFD amounted to 25.0% (2/8).

## DISCUSSION

We analyzed plasma levels and clinical data from 29 patients with *FLT3*-mutated AML receiving concomitant midostaurin and posaconazole to evaluate potential DDI and their clinical impact. High inter- and intraindividual variability of midostaurin and posaconazole plasma exposure was observed. AEs were more frequent in our study population than in other studies and resulted in dose modification or interruption of midostaurin in seven patients.

The median terminal half-lives of midostaurin and its active metabolites CGP62221 and CGP52421 in plasma are approximately 20.9, 32.3, and 471 h (13). The mean apparent plasma clearance (CL/*F*) was 2.4–3.1 L/h in healthy subjects. In patients with AML and systemic mastocytosis, population pharmacokinetic estimates for clearance of midostaurin at steady state were 5.9 L/h and 4.4 L/h, respectively (13).

Previous studies have shown that strong CYP3A4 inhibitors significantly increase midostaurin plasma levels. In a pre-clinical pharmacokinetic study, co-administered with ketoconazole resulted in a 5.4-fold increase of midostaurin steady-state exposure (17). In a case series, four times higher minimum and maximum midostaurin plasma concentrations and areas under the curve were described when posaconazole was concomitantly used as prophylaxis compared to micafungin in patients with AML receiving chemotherapy with midostaurin (18). In a real-life pharmacokinetic interaction study in 35 patients, the magnitude of posaconazole inhibition was determined to be high by reporting a 5.1-fold accumulation of midostaurin at steady state during concomitant posaconazole intake compared to micafungin (19). In a post-hoc analysis of the RATIFY trial, concomitant administration of strong CYP3A4 inhibitors led to only a 1.44-fold increase in midostaurin plasma exposure compared to placebo (15). The geometric mean level of midostaurin in patients with concomitant strong CYP3A4 inhibitors was 1.9 mg/L, which was 3.18-fold lower compared to our cohort with a geometric mean of 6.05 mg/L (15). Interestingly, both studies concluded that concomitant administration of midostaurin and posaconazole does not aggravate safety issues despite increased midostaurin plasma levels, while this statement was not supported by clinical data or AE analysis (15, 19). We hypothesized that clearance of midostaurin is reduced due to CYP3A4 inhibition by posaconazole. Furthermore, confounding factors such as other interfering medications, exact sampling time points, among others, may be responsible for this finding.

In our study, adverse effects occurred in all patients; however, only 4% were attributed to DDI of concomitant use of midostaurin and posaconazole, whereby some of them could have been caused by midostaurin alone as well. Regarding the toxicity of midostaurin, the most frequently reported AEs in earlier studies were febrile neutropenia, petechia, epistaxis, lymphocytopenia, infections, pneumonia, dyspnea, and pleural effusion (1). A post-hoc analysis of the RATIFY trial showed that higher-grade AEs occurred sooner when midostaurin was given concomitantly to CYP3A4 inhibitors, but the absolute numbers of AEs were similar to the placebo group (15). The median midostaurin trough level of our patients with an AE with a DIPS score of ≥5 was 7.4 mg/L at the onset of this AE, slightly higher than the median of our full cohort and in the above-described analyses. We attributed eight cardiac adverse events in 5/29 patients to a DDI of midostaurin and posaconazole, with QTc prolongation being a recurring adverse event as described for other *FLT3* inhibitors (20). Cardiac side effects of midostaurin, such as QTc prolongation, sinus tachycardia, and pericardial effusion, should be closely monitored according to the recommendation of the manufacturer (13). In our cohort, more severe cardiac AEs, such as symptomatic bradycardia, perimyocarditis, and sudden cardiac death, occurred at a comparably high rate in 3/29 patients, considering that pharmacovigilance reports had reported such events very rarely before (21, 22). This underscores the importance of treatment risk stratification for cardiac diseases with serial electrocardiograms and echocardiography before and during treatment, as well as close collaboration with cardio-oncology service for patients with AML (23).

Incidence of probable and proven bIFD was 17.2%, markedly higher than in previous studies of patients with AML receiving antifungal prophylaxis (2, 24). Evidence on the incidence of IFD in patients receiving midostaurin is contradictory. Two retrospective studies reported no increased incidence of IFD (25, 26), while an Italian study reported a similar incidence of 20%, regardless of which antifungal prophylaxis was used (27). Median duration of neutropenia in our cohort was 19 days, being lower than in the above-mentioned studies (24 days without IFD vs. 25 days in the group with IFD [25]; and 22 days in patients with and without IFD [27]). This observation raises concerns about whether the DDI of midostaurin and posaconazole may compromise antifungal prophylaxis and eventually increase the risk for IFD. It is hypothesized that *FLT3* inhibition further hampers the immune response by decreasing the production of interferon-1 and the development of dendritic cells, both essential in the defense against IFD (28, 29). Immunological aspects related to midostaurin treatment to explain higher susceptibility to IFD remain to be elucidated in future studies.

### Limitations

Due to the observational nature of this study and the fact that posaconazole prophylaxis is a standard-of-care in patients with AML, a control cohort not receiving prophylaxis was not feasible. TDM was scheduled for weekdays only; therefore, time points of plasma levels in individual cycles differ. Only trough levels were collected; *C*_max_ of midostaurin was not determined. Drug levels of the active metabolites of midostaurin, CGP62221 and CGP52421, were not analyzed, and AEs were not recorded beyond midostaurin administration. Attribution of AEs using the DIPS score inherits potential bias due to the investigator’s judgment. This was a single-center analysis; thus, local confounders like incidence of IFD may limit the generalization of our results.

### Conclusion and outlook

In conclusion, the present explorative study showed that TDM during concomitant administration of midostaurin and posaconazole may be of benefit in case of suspected toxicity or missing treatment response. High inter- and intraindividual variability of plasma levels was measured, and a high rate of AEs for each patient was observed. Plasma levels in patients experiencing midostaurin-related AEs were significantly higher/lower than in those not having these findings. Discontinuation of midostaurin due to higher midostaurin plasma levels and clinically notable adverse events could negatively impact the prognosis of these patients, but data on outcomes need to be investigated in the future.

While treatment of AML becomes more diverse and individualized with the aim to harness an optimal tumor response while simultaneously minimizing toxicity, TDM may also support individualized dosing of *FLT3* inhibitors. There is an urgent need to establish TDM for midostaurin and its metabolites, including reference ranges validated according to response rate in patients with *FLT3*-mutated AML. The availability of TDM methods, as well as the establishment of a dose-exposure-response/toxicity, remains to be investigated (30). Based on our data, precise recommendations for dose adjustments when AEs occur cannot be given.

## Data Availability

On reasonable and approved requests made to the corresponding author, data can be shared through secure online platforms.

## SUPPLEMENTAL MATERIAL

Supplemental material aac.01951-25-s0001.docx Fig. S1 to S5; Tables S1 to S3.

ASM does not own the copyrights to Supplemental Material that may be linked to, or accessed through, an article. The authors have granted ASM a non-exclusive, world-wide license to publish the Supplemental Material files. Please contact the corresponding author directly for reuse.
