# Supplementary material for: Clinical impact of potential drug-drug interactions between midostaurin and posaconazole in FLT3-mutated AML
Source: Antimicrob Agents Chemother. 2026 May 12;70(6):e01951-25. doi: 10.1128/aac.01951-25 (PMC13231906; doi:10.1128/aac.01951-25)
Supplement: Supplemental material — Fig. S1 to S5; Tables S1 to S3. [file aac.01951-25-s0001.docx]

**Supplement Figure 1. Sampling plan**


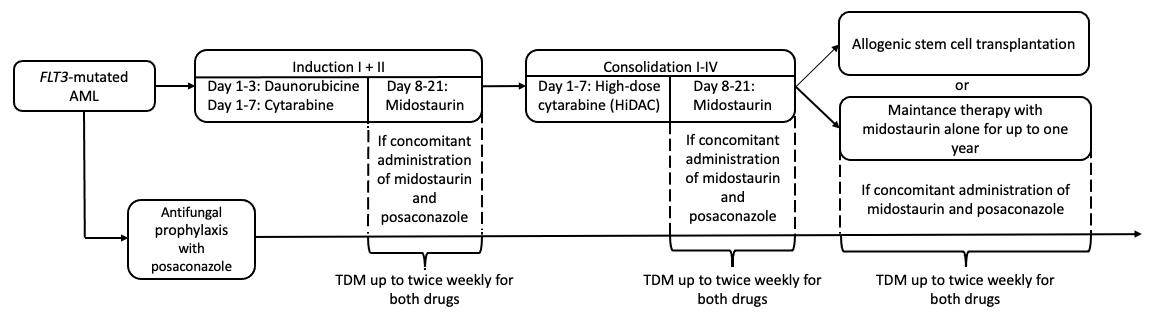


AML, acute myeloid leukemia; TDM, therapeutic drug monitoring

**Supplement Figure 2. CONSORT diagram**


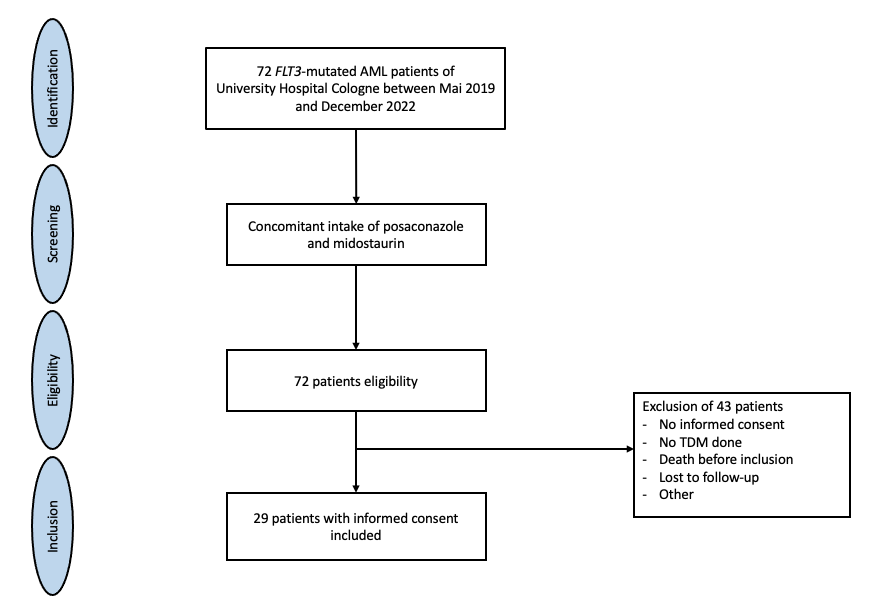


AML, acute myeloid leukemia; TDM, therapeutic drug monitoring

**Supplement Figure 3. Midostaurin and posaconazole plasma levels for each patient**

**Supplement Figure 4. Population pharmacokinetics (PPK) analysis : Goodness-of-fit plots**


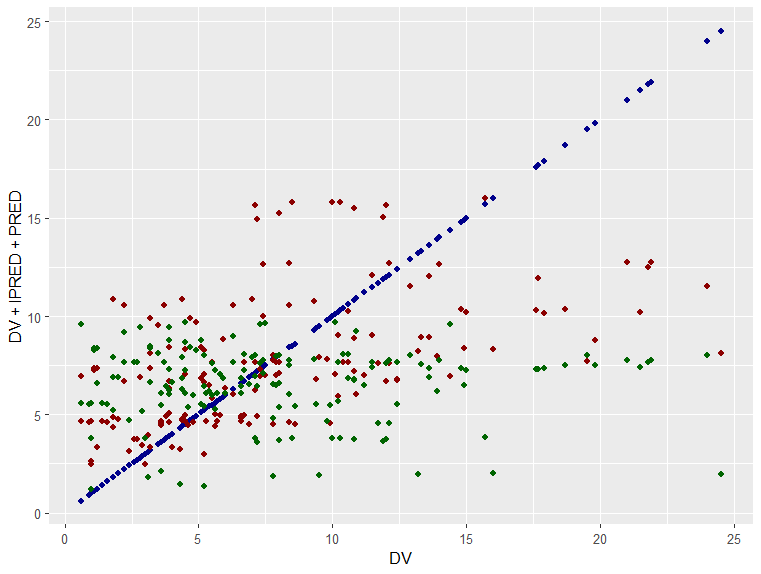


Green dots: PRED, predicted data (based on an overall clearance value); Red dots: IPRED, individual predicted data (based on an individual estimated clearance value); Blue dots: line of identity; DV, Data value

**Supplement Figure 5. Midostaurin and posaconazole plasma levels days before breakthrough IFD under posaconazole prophylaxis**

bIFD, Breakthrough invasive fungal disease under posaconazole prophylaxis

**Supplement Table 1. Concomitant medication other than posaconazole during midostaurin administration**

|  | Drug | Moderate or strong CYP3A4 inhibitor? | Indication | Dosage,  frequency,  administration | Total days of administration of other concomitant medication | Total days of simultaneously intake of midostaurin and other concomitant medication | TDM of other concomitant medication? | Further information |
| --- | --- | --- | --- | --- | --- | --- | --- | --- |
| Patient 1 | Ciprofloxacin | Moderate | Sepsis with *Klebsiella pneumoniae* | 200 mg q.i.d.  i.v. | 1 | 1 | No | Reanimation 7 days later, midostaurin and posaconazole were given concomitantly until 4 days before reanimation |
| Patient 2 | Ciprofloxacin | Moderate | Prophylaxis (neutrophil count below 1000) | 500 mg b.i.d.  p.o. | 7 | 3 | No |  |
| Patient 3 | Ciprofloxacin | Moderate | Prophylaxis | 500 mg b.i.d.  p.o. | 28 | 14 | No |  |
| Patient 4 | Ciprofloxacin | Moderate | Prophylaxis | 1 000 mg b.i.d. p.o. | 3 | 2 | No |  |
| Patient 5 | Clarithomycin | Strong | Beginning pneumonia | 250 mg b.i.d.  p.o. | 6 | 2 | No |  |
| Patient 6 | Voriconazole | Strong | Possible invasive fungal pneumonia (based on revised 2020 EORTC/MSG criteria) | 300 mg b.i.d.  p.o. | 22 | 22 | Day 12 after treatment start with voriconazole: 0.40 µg/ml  Day 22 after treatment start with voriconazole: 0.01 µg/ml | Posaconazole and voriconazole weren’t given concomitantly |
| Patient 7 | Voriconazole | Strong | Possible invasive fungal pneumonia (based on revised 2020 EORTC/MSG criteria) | 200 mg b.i.d.  p.o.,  later dosage was increased to 250 mg b.i.d. p.o. due to insufficient voriconazole plasma levels | 284 | 235 | Day 126 after treatment start with voriconazole: 0.2 µg/ml  Day 247 after treatment start with voriconazole: 0.5 µg/ml | Posaconazole and voriconazole weren’t given concomitantly |

i.v., intravenous; p.o., oral; q.i.d., four times a day; b.i.d., two times per day

**Supplement Table 2. Clinically notable grade 3 or higher infectious complications (n=82)**

| **Infectious complications with CTCAE >=3 (n=82)** | **n**  **[%]** | **Causative pathogen (n)** |
| --- | --- | --- |
| Febrile neutropenia | 35 [42.7] |  |
| Bacteremia   - Bacteremia catheter associated | 17 [20.7]  2 [2.4] | S. aureus (1), S. epidermidis (1), S. haemolyticus (2), MRSA (1), Gram-positive cocci (3), Streptococci (1), E. faecium (2), E. cloacae (1), E. gallinarium (1), E. coli (1), Enterococci (1), Enterococci and E. coli (1), Causative pathogen not identified (1) |
| Other infections that are device / catheter-related | 2 [2.4] | P. aeruginosa (1), Causative pathogen not identified (1) |
| Respiratory tract infections (bacterial, viral or fungal) | 12 [14.6] | IFD (8), COVID-19 (1), Legionella (1), Causative pathogen unknown (2) |
| Viral infections   - Viremia - Herpes simplex infection | 5 [6.1]  1 [1.2]  4 [4.8] | HSV viremia (1)  HSV 1 mucocutaneous infection (4) |
| Development of sepsis   - Neutropenic sepsis - Septic shock | 7 [8.5]  6 [7.3]  1 [1.2] | Causative agent of neutropenic sepsis unknown (6),  Septic shock due to grampositive cocci (1) |
| Other | 4 [4.8] | Neutropenic enterocolitis (2), Wound infection after ceasarian section (1), Clostridioides difficile infection (1) |

CTCAE, Common Terminology Criteria for Adverse Events; IFD, invasive fungal disease; MRSA, Methicillin-resistant Staphylococcus aureus; HSV, Herpes simplex virus

**Supplement Table 3. Case narratives for breakthrough IFD during posaconazole prophylaxis (n=1 proven, n=4 probable)**

| **Fungal infections**  **8/29** | **Site / type of infection** | **Microbiological findings** | **Treatment and outcome** | **Comment** |
| --- | --- | --- | --- | --- |
| 1.  *Rhizomucor pusillus* (proven) | Disseminated with lung and spleen involvement | Positive histology (Gomori silvering) and positive PCR from BAL and lung biopsy culture of lung biopsy negative | Combination therapy with liposomal amphotericin B and isavuconazole.  Patient survived and remained in complete remission until end of follow-up. | Breakthrough IFD, diagnosed eight days after stop of posaconazole prophylaxis; low plasma levels of posaconazole (627 µg/l) during prophylaxis. |
| 2.  *Aspergillus nidulans*  (probable) | Invasive pulmonary aspergillosis | Culture from BAL positive | Monotherapy with liposomal amphotericin B, then switch to voriconazole according to AFST.  Death one month after diagnosis of IFD due to progressive pulmonary GvHD. | Breakthrough IFD during posaconazole prophylaxis, no measurements of plasma levels done. |
| 3.  *Aspergillus species*  (probable) | Invasive pulmonary aspergillosis | Positive GM in BAL; culture and PCR from BAL negative | Monotherapy with liposomal amphotericin B, then switch to combination therapy of amphotericin B with ibrexafungerp.  After liver failure of unknown etiology, switch to monotherapy with caspofungin.  Death three days thereafter due to uncontrollable pneumonia and liver failure. | Breakthrough IFD during posaconazole prophylaxis with plasma level of 2027 µg/l nine days before diagnosis of IFD.  Midostaurin plasma levels were 24.5 mg/l and 16.0 mg/l four days and one day before diagnosis of IFD, respectively. |
| 4.  *Rhizopus species*  (probable) | Invasive pulmonary  infection | Rhizopus spp.: PCR from BAL positive  Aspergillus spp.: Positive GM in serum and BAL multiple times | Monotherapy with high-dose liposomal amphotericin B, then switch to combination with isavuconazole after three days.  Patient died seven days thereafter. | Breakthrough IFD during posaconazole prophylaxis, no measurements of plasma levels done. |
| 5.  *Trichosporon asahii*  (probable) | Invasive pulmonary  infection | Positive culture from sputum and bronchial secretion; positive GM in serum (cross-reactivity of Aspergillus and cryptococcal antigen | Antifungal monotherapy with liposomal amphotericin B.  Patient survived and was in complete remission until end of follow-up. | Breakthrough IFD during posaconazole prophylaxis. The patient received midostaurin for 13 days before diagnosis of IFD. No measurements of plasma level of both medications at the time of bIFD were done. |

IFD, invasive fungal disease; BAL, bronchoalveolar lavage; PCR, polymerase chain reaction; GvHD, graft-versus-host disease; GM, galactomannan antigen; AFST, antifungal susceptibility testing
